# Dermatomyositis with Anti-MDA5 Autoantibodies After SARS-CoV-2 mRNA Vaccination Treated with Tofacitinib: Integrating Literature Evidence and a Novel Observation

**DOI:** 10.3390/antib15020024

**Published:** 2026-03-09

**Authors:** Maurizio Benucci, Elisa Cioffi, Francesca Li Gobbi, Emanuele Antonio Maria Cassarà, Riccardo Terenzi, Edda Russo, Valentina Grossi, Barbara Lari, Maria Infantino, Mariangela Manfredi

**Affiliations:** 1 Rheumatology Unit, S. Giovanni di Dio Hospital, Azienda USL-Toscana Centro, 50134 Florence, Italy; maurizio.benucci@uslcentro.toscana.it (M.B.); elisa.cioffi@uslcentro.toscana.it (E.C.); francesca.ligobbi@uslcentro.toscana.it (F.L.G.); emanueleantonio.cassara@uslcentro.toscana.it (E.A.M.C.); riccardo.terenzi@uslcentro.toscana.it (R.T.); 2 Clinical Pathology S. Giuseppe Hospital, Azienda USL-Toscana Centro, 50053 Empoli, Italy; 3 Immunology and Allergology Laboratory Unit, S. Giovanni di Dio Hospital, Azienda USL-Toscana Centro, 50134 Florence, Italy; valentina2.grossi@uslcentro.toscana.it (V.G.); barbara.lari@uslcentro.toscana.it (B.L.); maria2.infantino@uslcentro.toscana.it (M.I.); mariangela.manfredi@uslcentro.toscana.it (M.M.)

**Keywords:** anti-MDA5-associated DM, dermatomyositis, mRNA vaccines (BNT162b2) (Pfizer/BioNTech), dermatomyositis, anti-MDA5, SARS-CoV-2 vaccination, tofacitinib

## Abstract

COVID-19 mRNA vaccines activate type I interferon pathways and in genetically or immunologically predisposed individuals may trigger autoimmune responses, including autoantibodies against melanoma differentiation-associated protein 5 (MDA5). Although cases of dermatomyositis (DM), particularly anti-MDA5-positive DM, have been increasingly reported after SARS-CoV-2 vaccination, its clinical spectrum and management remain incompletely defined. We conducted a narrative review of the literature on post-vaccination dermatomyositis, focusing on clinical features, autoantibody profiles, therapeutic approaches, and outcomes. The review was enriched by the inclusion of a new case: a 60-year-old woman who developed anti-MDA5-positive dermatomyositis two weeks after receiving her fourth dose of the BNT162b2 (Pfizer/BioNTech) vaccine. She presented predominantly with cutaneous and articular manifestations in the absence of interstitial lung disease. Treatment with oral prednisone, intravenous alprostadil, and the Janus kinase inhibitor tofacitinib resulted in marked clinical improvement. This case, together with the literature review, illustrates both typical and atypical presentations of vaccine-associated anti-MDA5 DM, highlights diagnostic challenges without lung involvement, and suggests JAK inhibition as a potential therapeutic option, contributing to a more comprehensive understanding of post-vaccination dermatomyositis.

## 1. Introduction

Dermatomyositis (DM) is a systemic autoimmune disorder characterized by both cutaneous and muscular involvement. Based on autoantibody profiles, DM can be classified into six distinct subgroups: anti-TIF1-γ, anti-NXP2, anti-MDA5 (melanoma differentiation-associated protein 5), anti-SAE (SUMO-activating enzyme 1), anti-Mi-2-associated, and autoantibody-negative DM, each associated with different prognostic implications [1]. Among these, anti-MDA5-positive DM is particularly notable for its strong association with interstitial lung involvement, including rapidly progressive interstitial lung disease (ILD/RP-ILD) and is associated with a high mortality rate [2].

In detail, MDA5 functions as a cytosolic sensor of long double-stranded RNA (dsRNA), initiating antiviral immune responses. Upon recognition of dsRNA, MDA5 activates mitochondrial antiviral-signaling protein (MAVS), which in turn triggers downstream signaling cascades involving TRIF (TIR domain-containing adaptor-inducing interferon-β), leading to the activation of nuclear factor κB (NF-κB) and interferon regulatory factor 3 (IRF3). Through these pathways, MDA5 promotes type I interferon production and contributes to inflammasome activation. In conjunction with LGP2 (ATP-dependent RNA helicase DHX58), MDA5 also serves as a key cytosolic sensor of SARS-CoV-2 infection [3,4,5].

Messenger RNA (mRNA) vaccines against COVID-19, including BNT162b2 (Pfizer/BioNTech) and mRNA-1273 (Moderna), have demonstrated high efficacy in reducing SARS-CoV-2 infection rates, disease severity, hospitalizations, and mortality [6]. These vaccines offer several advantages, including rapid development, low manufacturing cost, robust immunogenicity, and an excellent safety profile [7]. The most frequently reported adverse effects include local injection-site reactions and systemic symptoms such as fatigue, headache, myalgia, arthralgia, fever, and chills [8]. In addition, immunization against SARS-CoV-2 is particularly important for individuals with systemic rheumatic diseases (SRDs) who are at increased risk of severe outcomes following COVID-19 infection. However, it is important to note that patients with SRDs were largely excluded from pivotal clinical trials of mRNA vaccines [9].

Emerging evidence suggests that mRNA-based COVID-19 vaccines may induce type I interferon (IFN) responses, which in rare instances could lead to the generation of autoantibodies targeting MDA5 [10]. The potential clinical significance of anti-MDA5 autoantibodies in vaccine-associated DM has been described in several case series [11,12]. Moreover, rare instances of myocarditis and pericarditis, predominantly in male adolescents and young adults, have also been reported following administration of mRNA vaccines [13]. Current hypotheses propose that mRNA vaccines could, in rare cases, precipitate disease flares or de novo SRDs through mechanisms such as molecular mimicry, nonspecific immune activation, or other adjuvant effects.

Mechanistically, MDA5 functions as an intracellular viral sensor recognizing diverse viral RNA species and activating type I IFN production, which exerts antiviral effects while further upregulating MDA5, establishing a positive feedback loop [14,15,16]. Integrated miRNA–mRNA analyses in circulating monocytes from MDA5-positive diabetes mellitus patients reveal that TLR3, TLR7, and TLR9—sensors of viral nucleic acids—are transcriptionally regulated by PU.1, amplifying type I IFN-driven inflammation and promoting CCL2 secretion [17]. These data suggest that viral infections, including single- and double-stranded RNA viruses, may act as environmental triggers for MDA5 autoimmunity. Although MDA5 preferentially recognizes picornaviruses, additional evidence implicates HSV-1 and enterovirus B, supported by gene set enrichment analyses in dermatomyositis-associated ILD [18] and increased antibody reactivity against enterovirus B peptides [19].

The integration of mechanistic and clinical evidence supports a model whereby vaccination could transiently activate MDA5-related innate antiviral pathways in susceptible individuals, potentially unmasking or triggering autoimmune manifestations. Anti-MDA5 autoantibodies are strongly associated with clinically amyopathic dermatomyositis (CADM), characterized by classic skin findings without overt muscle weakness. Epitope mapping identifies the helicase domain as immunodominant [20,21], with population-specific differences: Japanese patients’ antibodies preferentially target CARD fragments, whereas American patients’ antibodies favor the C-terminal domain (CTD) [22]. Epitope specificity correlates with clinical phenotype and prognosis, with CTD-directed antibodies more frequent in females, helicase-directed antibodies associated with muscle or vascular involvement [23], and CARD-directed antibodies linked to rapidly progressive ILD.

This study aims to review the available literature on anti-MDA5-positive dermatomyositis occurring after SARS-CoV-2 vaccination, with particular focus on clinical features and therapeutic approaches, and to report our case of anti-MDA5-positive dermatomyositis developing shortly after the fourth dose of the BNT162b2 SARS-CoV-2 mRNA vaccine, in the absence of interstitial lung disease, with a favorable clinical response to treatment with the JAK inhibitor tofacitinib.

## 2. Review of the Literature

### 2.1. Search Strategy and Selection Criteria

A narrative review of the literature was performed in accordance with MDPI guidelines. PubMed/MEDLINE was searched to identify articles reporting cases of dermatomyositis following SARS-CoV-2 vaccination. The search strategy included combinations of the following terms: “dermatomyositis,” “anti-MDA5,” “COVID-19 vaccine,” “SARS-CoV-2 vaccination,” and “mRNA vaccine.” Only articles published in English were considered. Case reports and case series describing new-onset dermatomyositis temporally associated with COVID-19 vaccination were included. Reviews without original data, experimental studies, and reports lacking sufficient clinical information were excluded. The reference lists of selected articles were manually screened to identify additional relevant publications.

### 2.2. Main Reported Cases of MDA5 Dermatomyositis Following SARS-CoV-2 Vaccination

Several reports have described new-onset DM occurring after SARS-CoV-2 vaccination, predominantly following mRNA vaccine platforms.

A case report published in October 2021 suggested a potential link between COVID-19 vaccination and the onset of MDA5-DM [24]. In that case, a 58-year-old man developed respiratory failure and shock four days after receiving an mRNA COVID-19 vaccine, although the vaccine type and whether symptoms occurred after the first or second dose were not specified by Carrasco et al. [11]. Notable clinical features included oral blisters, digital ischemia and ulceration, and diffuse subpleural ground-glass opacities (GGO) on chest computed tomography (CT). An extensive infectious workup, including testing for COVID-19, was negative, and the diagnosis was confirmed by the presence of serum anti-MDA5 antibodies. This report represents the first clinical evidence supporting a potential relationship between COVID-19 vaccination, MDA5 activation, and subsequent disease development. In a case-based review focused on anti-MDA5-positive disease, Gonzalez et al. identified multiple cases of dermatomyositis developing shortly after COVID-19 vaccination, highlighting characteristic cutaneous findings and autoantibody positivity in patients receiving various vaccines [11]. Six initial cases involved rapid onset of rashes and systemic manifestations within days of vaccination. Notable examples include: a 45-year-old Hispanic male developing widespread rashes, Gottron’s papules, heliotrope rash, V-sign, Shawl sign, and erosive lesions after the second Moderna dose, with hyperferritinemia and high-titer anti-MDA5 antibodies, complicated by hypoxia and requiring IVIG, steroids, and rituximab, later stabilized on methotrexate; a 58-year-old Asian female who developed facial and knee rashes progressing to severe polyarthralgia, dyspnea, and interstitial lung disease after the second Covishield dose, treated sequentially with multiple immunosuppressants including rituximab, tofacitinib, and IVIG, with eventual stabilization; a 45-year-old Caucasian female with rheumatoid arthritis developing mechanic hands, fingertip ulcerations, and bilateral GGOs after the second Pfizer dose, managed with rituximab, high-dose steroids, plasma exchange, and tofacitinib; a 28-year-old Caucasian female with hypothyroidism and Brooke–Spiegler syndrome who developed widespread cutaneous rashes and proximal muscle weakness after the second Pfizer dose, treated with hydroxychloroquine, prednisone, and mycophenolate mofetil, remaining stable without lung involvement; a 51-year-old Asian female with prior COVID-19 infection who developed photosensitive rashes, polyarthralgia, and rapidly progressive ILD one week after her second Pfizer dose, treated with high-dose steroids and cyclophosphamide; and a 54-year-old Hispanic female with prior mild COVID-19 infection who developed burning rashes, facial edema, and polyarthralgia following the first Pfizer dose, worsening after the second dose, with strongly positive anti-MDA5 and anti-Ro52 antibodies, treated sequentially with azathioprine, mycophenolate mofetil, and methotrexate, achieving near-complete resolution of cutaneous symptoms. Collectively, these cases illustrate the spectrum of vaccine-associated anti-MDA5 dermatomyositis, ranging from amyopathic forms with isolated cutaneous involvement to rapidly progressive ILD, emphasizing the importance of early recognition, autoantibody testing, and tailored immunosuppressive therapy.

A case report of new-onset anti-MDA5 DM after the third dose of an mRNA vaccine described rash, fever, and UV-related skin ulcerations alongside high anti-MDA5 titers and CT abnormalities [25]. In detail, a 64-year-old female with asthma, allergic rhinitis, and prior mild COVID-19 infection developed progressive rash, facial and eyelid edema, Gottron’s papules, V-sign rash, and mild proximal weakness four weeks after her third Pfizer-BioNTech (BNT162b2) COVID-19 vaccine dose. Labs showed elevated CK, AST, ALT, ESR, CRP, ferritin, and strongly positive anti-MDA5 and anti-Ro52 antibodies. Skin biopsy confirmed interface dermatitis. Chest CT revealed bilateral ground-glass opacities and interstitial thickening, while pulmonary function tests were normal. Malignancy and infections were ruled out. She was diagnosed with anti-MDA5 dermatomyositis with ILD and treated initially with methylprednisolone and methotrexate, later switched to azathioprine due to hepatotoxicity. Disease progression with skin ulceration prompted rituximab therapy (2 × 1000 mg) alongside ongoing immunosuppression. Two months later, rash and edema improved, ulcers persisted, and inflammatory markers normalized.

Another series, combining data on both MDA5 and other DM-associated autoantibodies (e.g., NXP2, Mi-2, TIF1γ), found that seven of seventeen new-onset DM cases occurred after mRNA vaccination, with typical skin and muscle manifestations and a range of autoantibody profiles [12]. The authors report four cases of dermatomyositis associated with MDA5 and/or NXP2 antibodies occurring after SARS-CoV-2 exposure. Three patients (two females, one male; ages 19–57) developed dermatomyositis 1–7 days after BNT162b2 vaccination, and one female patient developed disease 2 weeks after SARS-CoV-2 infection. Skin manifestations and proximal myalgia were present in all cases, with initial arthritis in two patients, severe dyspnea in one, and dysphagia in another. CK was elevated in two patients. Anti-MDA5 antibodies were detected in three patients, NXP2 in two (one patient positive for both). Muscle MRI in three patients showed bilateral proximal myositis; skin and muscle biopsies confirmed dermatomyositis. One patient developed rapidly progressive ILD. All required immunosuppression with glucocorticoid pulses. Patients with milder symptoms were treated with hydroxychloroquine and azathioprine, while two critically ill patients required prolonged hospitalization and intensive immunosuppression including ciclosporin A, mycophenolate mofetil, and rituximab. Notably, new-onset dermatomyositis cases in their center increased during the pandemic, from 0.06–0.15% (2017–2020) to 0.26% in 2021.

In addition to anti-MDA5-positive disease, broader observational data indicate that new-onset or flares of DM may arise following SARS-CoV-2 vaccination, though causality remains unproven [26]. In detail, Chan et al. presented a single-institution retrospective case series assessing DM onset or exacerbation following SARS-CoV-2 vaccination. Among 53 patients with established or newly diagnosed DM, the authors identified two new cases of DM and three disease flares occurring within 1–30 days after COVID-19 vaccination, representing approximately 5.9% of the cohort. Onset or exacerbation was defined by increased fatigue, muscle soreness/weakness, or worsening cutaneous signs such as heliotrope rash or Gottron’s papules. Notably, all patients with post-vaccine symptoms had normal creatine kinase (CK) values, even when myositis was biopsy-confirmed. Most flares were mild and did not require changes in immunomodulatory therapy, while one new diagnosis necessitated hospitalization. The majority of the DM cohort did not report flares following vaccination. Although the small number of cases and the study design preclude causal conclusions, this series highlights that new-onset DM or disease exacerbation after SARS-CoV-2 vaccination may occur rarely, and proposes several immunologic mechanisms—such as vaccine-induced inflammatory responses—as possible contributors. Importantly, the authors emphasize that no definitive epidemiological link has been established, and their findings should be interpreted cautiously in the context of continued vaccination efforts. Table 1 summarizes the reported cases of dermatomyositis following SARS-CoV-2 vaccination.

### 2.3. Novel Case Observation of Vaccine-Associated Anti-MDA5 Dermatomyositis

A 60-year-old woman with a past medical history of well-controlled asthma presented to the Rheumatology Clinic (S. Giovanni di Dio Hospital, Azienda USL-Toscana Centro, Florence, Italy) with a suspected diagnosis of DM following administration of the fourth dose of the BNT162b2 mRNA COVID-19 vaccine (Pfizer/BioNTech). She had received the first vaccine dose on 1 June 2021, followed by subsequent doses on 16 October 2022, 19 November 2023, and 14 November 2024. Approximately two weeks after the last dose, she developed an erythematous, burning rash on the lateral aspect of her left arm, which progressively extended to the neck, face, anterior chest, and later to the metacarpophalangeal (MCP) joints. Ulcerative lesions appeared on the fingertips. Additional symptoms included bilateral periorbital and facial edema, hyperkeratotic eruptions on the lateral surfaces of the fingers, and polyarthralgia predominantly involving the hands and knees.

Due to the suspicion of a vaccine-related cutaneous reaction, she was initially treated with oral prednisone 25 mg/day, resulting in partial symptomatic improvement. However, over the next two months her cutaneous symptoms worsened, prompting rheumatologic evaluation.

**Figure 1:**
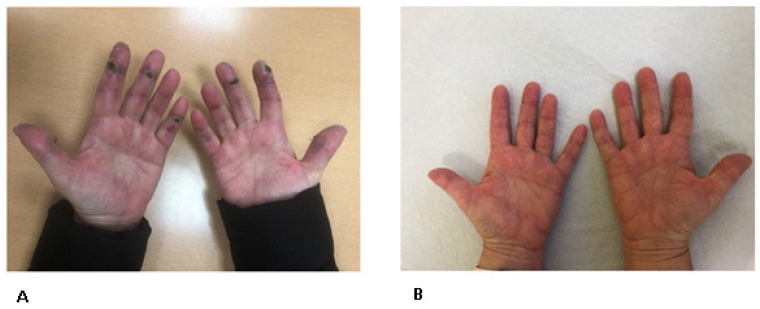
Clinical appearance of hyperkeratotic hands before treatment (**A**) and after treatment (**B**).

Physical examination revealed marked erythema of the forehead and cheeks (sunburn sign), violaceous discoloration of the eyelids (heliotrope rash), erythema over the upper chest (V-sign), Gottron’s papules over the MCP joints, periungual erythema, and hyperkeratotic “mechanic’s hands” (Figure 1A,B). There was no evidence of synovitis or muscle weakness. Cardiopulmonary examination was unremarkable.

Laboratory evaluation showed a normal complete blood count, urinalysis, and renal and thyroid function tests. Mild elevations in AST = 35 (NV < 32 U/L) and LDH = 234 (NV < 214 U/L) were observed, while ALT = 29 (NV < 52) was normal, and CPK = 64 U/L (NV < 180) and aldolase = 4.4 U/L (N < 7.6) were within normal limits. Inflammatory markers were elevated, with CRP = 1.61 mg/dL (NV < 0.50) and ESR = 26 mm/h (NV < 20). Complement C3, C4, and total immunoglobulin G levels were normal.

However, during the 24 h wait in the Emergency Room before admission to the Rheumatology Unit, the patient’s urgent tests showed C-reactive protein (CRP) 1.2 (<0.5 mg/dL), creatine kinase (CK) 236 (<194 U/L), aspartate aminotransferase (AST) 66 (<40 U/L), alanine transaminase (ALT) 139 (<50 U/L), lactate dehydrogenase (LDH) 485 (300–450 U/L), myoglobin 115 (<85 ng/mL), and, evaluated for possible myositis, she was treated with methylprednisolone 125 mg intravenously.

Antinuclear antibodies (ANAs) were positive at a titer of 1:640, displaying a fine-dotted nuclear pattern (AC 4-5-31, AC 8-9-10). Tests for anti-dsDNA, anti-Sm, anti-RNP, and anti-La/SS-B antibodies were negative. Myositis-specific and myositis-associated antibodies, assessed by a commercial line blot assay (EUROLINE Autoimmune Inflammatory Myopathies; Euroimmun, Lübeck, Germany), revealed positivity for anti-MDA5 = 54, (N < 20 CU/mL) and anti-Ro-52 = 23 (N < 10) antibodies.

Computed tomography scans of the chest, abdomen, and pelvis did not demonstrate interstitial lung disease or occult malignancy. Electromyography and muscle biopsy were not performed. Based on the characteristic cutaneous findings, absence of muscle involvement, and the presence of anti-MDA5 and anti-Ro-52 antibodies, a diagnosis of amyopathic dermatomyositis was established, temporally associated with recent COVID-19 vaccination.

The patient was treated with monthly alprostadil infusions (5 days per month for six months) for digital ulcers, oral prednisone (25 mg/day, gradually tapered), and tofacitinib (10 mg/day). At the most recent follow-up in June 2025, she demonstrated a marked clinical response, with near-complete resolution of facial edema and cutaneous lesions (Table 2).

### 2.4. Comparison with Previously Reported Post-Vaccination Anti-MDA5 Dermatomyositis Cases

A critical analysis of available reports reveals significant divergences in published cases of post-SARS-CoV-2 vaccination dermatomyositis, particularly in anti-MDA5-positive disease. Cutaneous manifestations are the most consistent feature, including heliotrope rash, Gottron papules, V-shaped sign, shawl sign, mechanic’s hands, and digital ulcerations, often with features associated with vasculopathy with digital ischemia. In contrast, systemic involvement varies greatly: while a substantial percentage of anti-MDA5-positive patients develop ILD, sometimes rapidly progressive, a minority present with amyopathic or skin-predominant forms without significant elevations of muscle enzymes or pulmonary involvement. Disease onset is typically acute, occurring within a few days to two weeks of vaccination, most commonly after the first or second mRNA dose, with cases occurring after subsequent doses being decidedly rare. From an immunological perspective, anti-MDA5 antibodies, often associated with anti-Ro52 antibodies, are overrepresented in severe phenotypes, particularly those with pulmonary involvement, although larger series confirm the overall heterogeneity of autoantibodies. Laboratory and imaging findings correlate with clinical severity: hyperferritinemia and elevated inflammatory markers are more common in systemic disease, ground-glass opacities assessed by CT scan are frequent in severe cases, while elevated muscle enzymes do not always correlate, especially in amyopathic presentations.

Our new case exemplifies vaccine-associated amyopathic DM with prominent skin manifestations without systemic involvement and represents the first case reported after a fourth dose of vaccine, broadening the spectrum of post-vaccination anti-MDA5 DM. Previous case series have documented onset mainly after the first or second dose [27,28], with rare reports after three doses [29,30]; a retrospective cohort identified four cases after the first dose of RNA vaccines [31]. This case contrasts with previously reported cases in several key aspects such as the absence of ILD despite anti-MDA5 positivity and disease onset after the fourth dose of BNT162b2 rather than the first or second. In detail, published studies have shown that in anti-MDA5+ patients (n = 83).

In detail, published studies have shown that within anti-MDA5+ patients (n = 83), three subgroups have been identified. One group (18.1%) corresponded to patients with rapidly progressive ILD (93.3%; *p* < 0.0001 for all) and a very high mortality rate. The second subgroup (55.4%) corresponded to patients with pure dermato-rheumatologic symptoms (arthralgia; 82.6%; *p* < 0.01) and a good prognosis. The third corresponded to patients, predominantly male (72.7%; *p* < 0.0001), with severe cutaneous vasculopathy, frequent signs of myositis (proximal weakness: 68.2%; *p* < 0.0001), and an intermediate prognosis. Raynaud’s phenomenon, arthralgia/arthritis, and gender allowed for cluster membership (83.3% correct estimate). Therefore, we can affirm that the absence of the ILD appearance places our case in the second phenotype [32].

In addition, the data in the literature on the timing are different, however systematic reviews of case reports indicate an onset of the DM picture from 3 days up to two months after vaccination [11]. The potential role of innate antiviral signaling pathways in linking MDA5-positive DM to mRNA vaccination has been the focus of recent investigations. Anti-MDA5 antibodies have been proposed as predictors of severe disease progression with ILD following mRNA COVID-19 vaccination, and there is also emerging evidence suggesting a possible association between anti-MDA5 positivity and post-vaccination paraneoplastic DM [30]. An additional noteworthy aspect of the present case is that disease onset occurred after the fourth vaccine dose—this appears to be the first such reported instance. Previous case series have documented onset primarily after the first and second vaccine doses: in one series of six cases, five developed DM after the second dose and one after the first dose [11].

### 2.5. Treatment of Anti-MDA5 Dermatomyositis: From Corticosteroids to JAK Inhibitors

Therapeutic strategies for dermatomyositis occurring after SARS-CoV-2 vaccination largely mirror those adopted for idiopathic dermatomyositis and are tailored according to disease severity and organ involvement. They include corticosteroids, conventional immunosuppressants, calcineurin inhibitors, high-dose IVIG, and biologics such as rituximab.

Systemic corticosteroids represent the mainstay of treatment and have been used as first-line therapy in most reported cases, often achieving partial or complete clinical improvement [33,34]. In patients with inadequate response or more severe disease, additional conventional immunosuppressive agents—including methotrexate, mycophenolate mofetil, azathioprine, tacrolimus, and cyclophosphamide—have been employed, particularly in cases complicated by interstitial lung disease [33,34,35,36].

Anti-MDA5-positive dermatomyositis frequently requires a more aggressive approach due to its association with severe cutaneous involvement and an increased risk of rapidly progressive interstitial lung disease. Published reports indicate that treatment intensity is primarily driven by clinical phenotype and organ involvement: patients with limited cutaneous or amyopathic presentations may respond to moderate immunosuppression, whereas those with pulmonary involvement often require early combination regimens [34,37]. Given the interferon-driven pathogenesis of anti-MDA5 dermatomyositis, Janus kinase (JAK) inhibitors—particularly tofacitinib—have emerged as a promising option in refractory or severe disease, based on their ability to inhibit type I interferon signaling [37,38,39]. Although current evidence is limited to case reports and small case series, available data suggest that JAK inhibitors may improve pulmonary, cutaneous, and articular manifestations with a generally acceptable safety profile in selected patients; however, the potential risk of infectious complications necessitates careful patient selection and close monitoring.

### 2.6. Targeted and Phenotype-Adapted Therapy in Anti-MDA5 Dermatomyositis

Therapeutic strategies for anti-MDA5-positive dermatomyositis, including cases following SARS-CoV-2 vaccination, are largely dictated by disease severity and organ involvement. In general, treatment intensity mirrors the clinical phenotype: patients with ILD often require early high-dose glucocorticoids in combination with conventional immunosuppressants such as cyclophosphamide, calcineurin inhibitors, methotrexate, mycophenolate mofetil, or azathioprine, and in some instances biologic agents like rituximab [33,34,35,36,37] Conversely, patients with milder or predominantly cutaneous disease frequently achieve stabilization or improvement with moderate immunosuppression. The aggressive nature of anti-MDA5-positive disease, particularly when associated with severe cutaneous involvement or rapidly progressive ILD, underscores the need for tailored therapeutic approaches [34,37].

Emerging evidence highlights the role of Janus kinase (JAK) inhibitors as a targeted therapy grounded in the interferon-driven pathogenesis of anti-MDA5 dermatomyositis. JAK inhibitors have recently gained interest in MDA5-positive DM because they directly target the type I IFN signaling pathway, which, as reported, plays a central role in disease pathogenesis. In addition, JAK inhibition affects type II IFN and IL-21 signaling—pathways that contribute to tissue fibrosis—via the JAK-STAT axis, specifically through JAK1–STAT1 and JAK3–STAT5 signaling [40,41]. Consequently, JAK inhibitors may attenuate acute inflammation and reduce progression to chronic pulmonary fibrosis in this patient population. Activation of type I interferon pathways contributes to both cutaneous and pulmonary manifestations, providing a strong rationale for JAK inhibition in refractory cases [23,27,38,39,40,41,42,43,44,45,46].

Clinical reports and small case series demonstrate improvement in pulmonary, cutaneous, and articular features—including ulcerative skin lesions and arthritis—that are often poorly responsive to conventional therapies [39,43,44]. Although JAK inhibitors are most frequently administered in combination with conventional triple therapy in refractory cases, a recent meta-analysis of 58 patients suggests that their clinical efficacy may be independent of the timing of initiation [28]. Dose escalation has been explored in severe, treatment-resistant cases, yielding clinical benefit in a subset of patients, though with increased infectious risk [42]. Therapeutic benefits have been reported across multiple JAK inhibitor classes, including tofacitinib, upadacitinib, and baricitinib, suggesting a class effect rather than a drug-specific effect [26,29,46,47].

Outcomes in published cases are closely tied to phenotype and organ involvement. While patients with limited or cutaneous-dominant disease typically respond to moderate immunosuppression, those with ILD require intensive combination therapy. Rapidly progressive ILD cases demand aggressive management but are not uniformly fatal. In our patient, the predominance of cutaneous and articular manifestations supported the use of a JAK inhibitor, targeting type I interferon as well as type II IFN and IL-21 pathways implicated in tissue fibrosis via the JAK–STAT axis [27,36,37,44]. Collectively, these observations support a severity-adapted therapeutic approach in anti-MDA5 dermatomyositis, with growing consideration of JAK inhibition in interferon-driven disease, while emphasizing the need for careful patient selection, vigilant monitoring for infectious complications, and further prospective studies to optimize timing, safety, and long-term outcomes.

## 3. Conclusions

In conclusion, post-vaccination anti-MDA5 DM appears to be a rare but clinically significant entity characterized by heterogeneous cutaneous, muscular, and systemic manifestations. Integration of published cases with our original observation highlights both shared features—such as typical skin findings and anti-MDA5 positivity—and variability in timing of onset, extent of muscle and pulmonary involvement, and autoantibody patterns. Although a causal relationship cannot be definitively established, current mechanistic insights into MDA5-mediated innate antiviral signaling and type I interferon pathways provide a biologically plausible framework linking vaccination to disease onset in susceptible individuals.

Early recognition, comprehensive autoantibody assessment, and phenotype-adapted immunosuppressive therapy—including consideration of targeted approaches such as JAK inhibition in selected cases—are essential to optimize outcomes. Ongoing vigilance, systematic reporting, and further mechanistic and prospective studies are warranted to better define risk factors, clarify potential associations such as severe ILD progression or paraneoplastic presentations [29], and refine therapeutic strategies in this setting.

## Figures and Tables

**Table 1:** Reported cases of dermatomyositis following SARS-CoV-2 vaccination.

Study (Ref.)	No. of Anti-MDA5 Cases	Vaccine/Dose	Time to Onset	Key Clinical Features	ILD	Treatment	Outcome
Gonzalez et al., 2022 [11]	6	Moderna (2nd), Pfizer (1st/2nd), Covishield (2nd)	Days–1 week	Classic DM rashes (Gottron’s papules, heliotrope rash, V-sign, Shawl sign), digital ulcers, polyarthralgia; hyperferritinemia; anti-MDA5+ (some anti-Ro52+)	Variable (none to RP-ILD)	IV steroids, IVIG, rituximab, tofacitinib, MTX, MMF, cyclophosphamide	Stabilization to partial/near remission
Holzer et al., 2022 [12]	3 anti-MDA5 (of 4 MDA5/NXP2)	Pfizer (BNT162b2)	1–7 days	Age 19–57; rash + proximal myalgia; arthritis (2), severe dyspnea (1), dysphagia (1); CK↑ (2); biopsy/MRI consistent with DM	1 RP-ILD	Steroid pulses; HCQ, AZA; severe cases: cyclosporine A, MMF, rituximab	Variable; severe cases required hospitalization
Carrasco et al., 2021 [24]	1	mRNA (type not specified)	4 days	58-year-old male; oral blisters, digital ischemia/ulcers; diffuse subpleural GGOs; respiratory failure and shock; anti-MDA5+	Yes (RP-ILD)	Not fully detailed	Severe course; first reported case suggesting association
Bolla et al., 2024 [25]	1	Pfizer (BNT162b2), 3rd dose	4 weeks	64-year-old female; progressive rash, facial/eyelid edema, Gottron’s papules, V-sign; mild proximal weakness; CK↑; anti-MDA5+ and anti-Ro52+; bilateral GGOs	Yes	Methylprednisolone + MTX → azathioprine; rituximab	Clinical improvement; persistent ulcers
Chan et al., 2022 [26]	Not specified (DM overall)	Various	1–30 days	2 new-onset DM + 3 flares (of 53); fatigue, myalgia, worsening rash; normal CK even with biopsy-proven myositis	Not specified	Mostly no therapy change; 1 hospitalization	Mostly mild flares; no proven causality

**Table 2:** Clinical Characteristics, Laboratory Findings, and Treatment of the Present Case.

Study (Ref.)	No. of Anti-MDA5 Cases	Vaccine/Dose	Time to Onset	Key Clinical Features	ILD	Treatment	Outcome
Present case	1	Pfizer (BNT162b2), 4th dose	~2 weeks	60-year-old female; burning erythematous rash spreading to face/chest/MCP; heliotrope rash, Gottron’s papules, V-sign, mechanic’s hands, fingertip ulcers; polyarthralgia; ANA 1:640; anti-MDA5+ and anti-Ro52+; normal muscle enzymes initially; no weakness	No	Prednisone, alprostadil (for digital ulcers), tofacitinib	Marked clinical response; near-complete cutaneous resolution

## Data Availability

The patient data is contained in their medical records.

## Abbreviations

DM	dermatomyositis
ILD	interstitial lung disease
IS	immunosuppressants
MTX	methotrexate
NR	not reported.3.3 Anti-MDA5-positive cases after COVID-19 vaccination
